# UAV-Based Road Feature Database Construction for Traffic Engineering: A Three-Layer Framework, Freeway and Urban Applications, and Emerging Frontiers

**DOI:** 10.3390/s26216751

**Published:** 2026-10-25

**Authors:** Kan Gu, Yizhe Wang, Zheng Yang, Jie Yang

**Affiliations:** 1 Hangzhou Institute of Communications Planning Design & Research Co., Ltd., No. 8 Jiande Road, Hangzhou 310006, China; hzjtgh@163.com; 2 The Key Laboratory of Road and Traffic Engineering, Ministry of Education, Tongji University, No. 4800 Cao’an Road, Shanghai 201804, China; yj22@tongji.edu.cn; 3 School of Naval Architecture Ocean and Civil Engineering, Shanghai Jiao Tong University, No. 800 Dongchuan Road, Shanghai 200240, China; daredevildd@sjtu.edu.cn

**Keywords:** unmanned aerial vehicle, road feature database, trajectory dataset, traffic engineering, computer vision, multi-object tracking

## Abstract

**Highlights:**

**What are the main findings?**

- Read from a database-centric perspective, roughly 70 UAV traffic studies yield a three-layer reference framework—static infrastructure, dynamic road-user, and derived semantic features—and a six-stage construction workflow that no existing survey of UAV traffic sensing provides.
- Aerial perception accuracy is no longer the binding constraint; validation against reference sensors, standardization, and coverage of data-poor regimes (long durations, adverse weather, night, and underrepresented regions) now dominate the research agenda.

**What are the implications of the main findings?**

- Co-registered static–dynamic databases can convert one-off drone campaigns into reusable engineering assets for safety analysis, operations, simulation calibration, and digital twins.
- Remote sensing foundation models, cooperative perception, and federated learning offer concrete pathways toward scalable, privacy-aware database construction under the emerging low-altitude economy.

**Abstract:**

Unmanned aerial vehicles (UAVs) have matured into a credible sensing layer for traffic engineering, yet the knowledge they generate remains scattered across one-off campaigns, bespoke pipelines, and scenario-specific studies. Existing surveys treat the UAV as a measurement instrument or organize the field by task, leaving unaddressed what becomes of the observations after the flight. This review takes the complementary, database-centric perspective and asks how UAV-derived observations can be consolidated into reusable road feature databases. Drawing on approximately 70 studies published between 2017 and 2026, it makes five contributions: (1) a sensing-oriented appraisal of the aerial perception chain—detection, tracking, stabilization, and georeferencing—judged by downstream trajectory quality and by validation against reference sensors; (2) a three-layer reference framework of static infrastructure, dynamic road-user, and derived semantic features bound by co-registration and provenance; (3) a six-stage construction workflow that makes the key implementation issues and remaining gaps at each stage explicit; (4) a regime-based comparison of continuous-flow freeway and interrupted-flow urban applications, with a consolidated landscape of twelve trajectory dataset releases that exposes data-poor regimes; and (5) a cross-cutting synthesis of eleven research streams, four technological frontiers (remote sensing vision–language models, large language model agents, cooperative perception, and federated learning) with the low-altitude economy as their institutional substrate, and the operational preconditions of sustained observation, distilled into six challenge clusters with priority directions. Perception accuracy is no longer the binding constraint; standardization, ground-truth validation, coverage of underrepresented regimes, and closing the loop from observation to control now dominate the agenda.

## 1. Introduction

Traffic engineering has always been rationed by data. Inductive loops, microwave radar, and roadside cameras deliver point-based or short-range measurements, so the spatial evolution of maneuvers—merging, weaving, queue spillback, pedestrian–vehicle negotiation—must be inferred rather than observed. Unmanned aerial vehicles (UAVs) dissolve this constraint by providing a stable bird’s-eye view over hundreds of meters of roadway without instrumenting the infrastructure or alerting road users. A recent synthesis of more than 315 studies spanning 2009–2024 documents how UAV platforms coupled with artificial intelligence (AI) have improved traffic flow estimation accuracy by over 20%, enabled conflict analysis through surrogate measures such as time-to-collision (TTC), and supported tailored data collection protocols for facilities ranging from intersections to roundabouts [1]. That synthesis, however, treats the UAV primarily as a measurement instrument; the question of what happens to the data after the flight—whether observations accumulate into durable, queryable engineering assets—remains largely unaddressed, and it is precisely this question that motivates the present review.

Two converging forces make this the right moment to pose the database question. The first is institutional. Low-altitude economy policies are moving UAV operations from exceptional permissions toward routine airspace access, with recent policy analyses structuring the transition along dimensions of latency and resilience, autonomy, equipment, regulation, acceptance, coordination, and equity [2]. At the same time, infrastructure-inspection experience has cataloged the technical, legal, privacy, and liability barriers that still shape what can be flown, where, and how often [3]. The second force is demand-side. Digital twin (DT) research in road engineering finds that effort has concentrated on data perception and virtual model creation, while real-time physical–virtual interaction remains underexplored [4]; DT-oriented intelligent transportation system (ITS) reviews enumerate data fusion, cooperative perception, edge computing, and federated learning as enabling technologies awaiting reliable data supplies [5]; and infrastructure-focused surveys warn of fidelity–computation trade-offs and of the neglect of infrastructure health status [6]; both, we argue, presuppose adequate underlying data. In the control domain, a critical appraisal of urban signal systems argues that deployments over-rely on information technology while underusing traffic flow theory and the rich vehicle–infrastructure interaction data now becoming available [7]. All of these consumers presuppose exactly what current practice rarely delivers: structured, co-registered, reusable road data.

Between the supply of aerial pixels and this demand for engineering-grade data sits a gap that we characterize as the one campaign, one paper pattern. Perception papers optimize detectors and trackers on benchmark imagery; application papers mine a single flight campaign for a single facility and research question; datasets, where released, adopt bespoke formats, coordinate systems, and quality conventions. Computer vision surveys for ITS catalog an impressive breadth of applications—monitoring, incident detection, pricing, condition assessment—yet organize the field by task rather than by the consolidation of outputs [8]. This review takes the complementary, database-centric perspective. We define a road feature database as a co-registered repository with three layers: (i) static infrastructure features, including lane-level geometry, markings, boundaries, and pavement condition; (ii) dynamic road-user features, principally georeferenced trajectories with kinematics and interactions; and (iii) derived semantic features, such as surrogate safety indicators, calibrated behavioral parameters, and scenario libraries. The central claim developed throughout is that treating these layers as one asset—rather than as by-products of separate studies—changes what UAV research is for. The review is accordingly addressed to two readerships at once: the traffic engineers who consume the data, and the sensing researchers who design the platforms, sensors, and processing chains that produce it.

### 1.1. Scope, Sources, and Review Methodology

This article is a narrative review rather than a systematic one, and we state its construction transparently. The candidate literature was retrieved from Web of Science, Scopus, and IEEE Xplore, complemented by the proceedings of the flagship computer vision and intelligent transportation venues, using combinations of terms covering UAV or drone sensing, trajectory datasets, road feature extraction, traffic safety and operations, and the emerging-technology clusters examined in Section 6. The core window is 2017–2026. Studies were retained when they (a) contribute methods for converting aerial observation into road features, (b) release or substantially characterize datasets usable as database layers, (c) demonstrate traffic engineering applications of such data in freeway or urban scenarios, or (d) develop technologies with a credible, near-term bearing on database construction. Approximately seventy studies satisfied these criteria and are organized into seven thematic clusters: a motivating cluster drawn upon in this introduction, five that structure Section 2, Section 3, Section 4, Section 5 and Section 6, and a seventh, operational cluster on endurance, robustness, and mission planning that is drawn upon in Section 7.1 and Section 7.2. We deliberately favored archival journals and widely adopted benchmarks; a consequence, acknowledged here, is that gray literature and purely commercial systems are underrepresented.

### 1.2. Main Contributions and Novelty

The subject of this review is deliberately narrow, and it is worth stating what it is not. It is not a general survey of UAV traffic sensing, of which a recent and comprehensive example exists [1]; it is not a benchmark survey of aerial detection and tracking; and it is not an account of the low-altitude economy at large. Its subject is the construction of reusable road feature databases from UAV observations, and its central question is how the outputs of individual flights, pipelines, and studies can be consolidated into a co-registered, layered, and maintained engineering asset. To the best of our knowledge, it is the first review to treat the road feature database, rather than the UAV platform, the perception algorithm, or the individual application, as the unit of analysis.

Relative to the existing surveys of UAVs in traffic analysis [1], of computer vision in ITS [8], of UAV-based inspection [3], and of digital twins [4,5,6], this review makes five contributions. Each is delivered by a specific element of the manuscript, so that a reader can verify it directly:

(1) A sensing-oriented appraisal of the aerial perception chain (Section 2). Advances in detection, tracking, stabilization, and georeferencing are evaluated by their effect on downstream trajectory quality rather than by leaderboard position; the evidence on validation against independent reference sensors—probe vehicles equipped with high-precision satellite positioning and inertial sensors, induction loops, and in-vehicle smartphone sensors—is consolidated; and the complementary roles of aerial observation and onboard inertial sensing are articulated.
(2) A three-layer reference framework for road feature databases (Section 3.1, Section 3.2, Section 3.3 and Section 3.4). Static infrastructure, dynamic road-user, and derived semantic features, which the literature has so far developed in isolation, are integrated under a common discipline of co-registration and provenance, and the framework is shown to match the layered data architectures that engineering platforms have arrived at independently (Section 6.3).
(3) A six-stage construction workflow (Section 3.5). Distilled from the reviewed pipelines and campaigns, the workflow runs from acquisition design through perception, georeferencing, quality assurance, and schema design to release and maintenance, and makes explicit the key implementation issues, the most instructive practices, and the remaining gap at each stage. No existing survey specifies how a road feature database is built.
(4) A regime-based comparison of applications and datasets (Section 4 and Section 5). Continuous flow on freeways is contrasted with interrupted flow on urban streets, an organizing device borrowed from capacity analysis, and twelve trajectory dataset releases are consolidated into a single landscape; the comparison exposes which regimes remain data-poor in duration, geography, weather, and night conditions.
(5) A cross-cutting synthesis and research agenda (Section 6 and Section 7). Four technological frontiers and the low-altitude economy are appraised through the single lens of what each changes for database construction; eleven research streams are connected by methodological family, data basis, and evaluation metrics; the chain along which errors propagate, the research lineages within which results are comparable, and three technological waves are identified; five operational preconditions of sustained observation are set out; and six challenge clusters are paired with enabling assets and priority directions.

Three features of these contributions speak directly to sensing research, which is the second readership of this review. First, the UAV is treated as a sensing system whose output must meet application-defined specifications: the review traces how the acquisition parameters that a flight team controls—altitude, and with it ground sampling distance and field of view; stabilization; georeferencing; clock synchronization across aircraft; and session length under battery endurance—propagate into the positional, speed, and acceleration fidelity of the resulting trajectories and into the detectability of lane markings and pavement distress (Section 2.3, Section 3.3, Section 3.5 and Section 7.2). Second, it consolidates the evidence on validation against independent reference sensors and articulates the complementary roles of aerial observation and onboard inertial and satellite positioning (Section 2.3), a synthesis that neither [1] nor [8] provides. Third, it identifies where sensing rather than analysis is the binding constraint: pavement distress detection and image–LiDAR fusion whose value depends on sensor quality (Section 3.3), and adverse-weather and night operation and endurance (Section 7.2). In this sense the review is application-driven: the traffic engineering uses of Section 4 and Section 5 define the specifications that the aerial sensing chain of Section 2 and Section 3 must satisfy.

Table 1 positions the review against the surveys with which it shares the most ground, using the five contributions as the dimensions of comparison. The UAV-centered survey of Obaid et al. [1] and the computer-vision survey of Dilek and Dener [8] cover the perception side more broadly than we do, but organize their material by facility or by task and stop at the point where observations become data; the inspection-oriented review of Adibfar et al. [3] catalogs the technical and legal conditions of flying without asking what becomes of the imagery; and the digital twin surveys [4,5,6] start from the consumers of road data and treat its supply as given. None of the surveys compared in Table 1 treats the database as the object of study, specifies a construction workflow, contrasts continuous-flow and interrupted-flow applications as regimes with different data requirements, or traces the evaluation metrics of the field as a chain along which error propagates. Those elements are what this review adds. Throughout, we aim not merely to report who did what, but to compare, group, and evaluate: to say where the field has genuinely converged, where reported numbers should be read with caution, and where the next decade of effort is best invested.

### 1.3. Organization of the Review

Every section is organized around the central question stated above. Section 2 asks what the perception chain must deliver for the database to be trustworthy; Section 3 asks what the database contains and how it is built; Section 4 and Section 5 ask what it is used for in the two facility regimes of traffic engineering; Section 6 asks what will change its economics; Section 7 asks what connects the reviewed studies, what preconditions routine operation, and what still blocks it; and Section 8 concludes by returning to the five contributions and to the agenda they imply.

## 2. From Airborne Imagery to Traffic Data: The Aerial Perception Chain

Every road feature database inherits its quality ceiling from the perception chain that produced it: object detection, multi-object tracking (MOT), image stabilization, and georeferencing. This section reviews these stages with a deliberate emphasis on the aerial domain gap and, in keeping with the first contribution of Section 1.2, judges each stage by what it delivers to the database rather than by its benchmark rank. Progress on general-purpose benchmarks is a necessary input, but, as the evidence below shows, it is not a sufficient guarantee of engineering-grade trajectories; nadir viewpoints, tiny object footprints, platform motion, and long recording horizons impose failure modes that ground-level benchmarks never exercise.

### 2.1. Object Detection in Aerial Imagery

Two research streams feed aerial detection. The first is the general detector lineage. DETR reformulated detection as direct set prediction with bipartite matching, eliminating hand-crafted anchors and non-maximum suppression while matching a strong Faster R-CNN baseline on COCO [9]. Swin Transformer supplied a hierarchical backbone whose shifted-window attention achieves linear complexity in image size and lifted COCO detection to 58.7 box AP [10]. YOLOv9 revisited the information bottleneck in deep networks, introducing programmable gradient information (PGI) and the GELAN architecture so that models trained from scratch rival heavily pre-trained competitors [11]. These are genuine conceptual advances—set prediction, windowed attention, and gradient-information preservation, respectively—and all three ideas have diffused into aerial detectors.

The second stream confronts what makes drone imagery different. The VisDrone benchmark, collected across fourteen Chinese cities, institutionalized the task—with its drone-specific stressors of dense small objects, violent scale variation with altitude, and motion blur under high-speed low-altitude flight—while its organizers candidly document the cost of fully manual annotation [12,13]. Responses have been architectural. TPH-YOLOv5 added a fourth prediction head for minute objects, replaced the original heads with transformer prediction heads, and inserted convolutional attention, reaching 39.18% AP on the VisDrone2021 test-challenge split—about seven points above its YOLOv5 baseline and within a fraction of a point of the winning entry [13]. UAV-YOLO pursued the same goal through receptive-field engineering, restructuring residual blocks and shallow layers of YOLOv3 so that small targets retain spatial evidence [14]. The juxtaposition of benchmark regimes is instructive and, we would argue, under-appreciated: detectors that exceed 55 AP on COCO still plateau near 40 AP on VisDrone. Aerial detection is thus not a solved downstream application of mainstream vision; it remains a distinct regime in which architecture choices are ultimately trade-offs among accuracy, latency, and onboard deployability rather than pure accuracy contests.

### 2.2. Multi-Object Tracking Across Aerial Frames

Tracking-by-detection dominates aerial pipelines, and its evolution can be read as a debate about how much to trust appearance versus motion. DeepSORT augmented the original SORT association with a deep appearance metric learned offline on re-identification data, cutting identity switches by 45% and tolerating longer occlusions [15]. StrongSORT then showed that a carefully modernized DeepSORT—upgraded detection, embedding, and association, plus an appearance-free link module for global track completion and Gaussian-smoothed interpolation for missed detections, each costing only milliseconds—reaches state-of-the-art results across MOT17, MOT20, DanceTrack, and KITTI [16]. OC-SORT took the opposite stance: it retained a plain Kalman filter but corrected the error that accumulates during occlusion by re-anchoring on observations, remaining simple, online, and capable of more than 700 frames per second on a CPU while excelling precisely where motion is nonlinear [17]. For drone footage the balance tilts in an interesting way. The nadir view largely removes the occlusion that appearance cues were invented to survive, while tiny targets weaken those same appearance cues; motion-centric designs therefore age well at altitude. What the MOT literature has not yet internalized, in our reading, is that traffic engineering cares about a different loss function: a single identity switch inside a weaving segment corrupts lane change counts and conflict measures far more than its marginal effect on aggregate tracking scores suggests. Trajectory-level error reporting, rather than frame-level metrics alone, would make tracking research substantially more legible to its transportation consumers.

### 2.3. Stabilization, Georeferencing, and Trajectory Quality Assurance

The least glamorous stages of the chain turn out to discriminate most sharply between datasets that traffic engineers can trust and those they cannot. The HIGH-SIM effort extracted high-granularity trajectories from helicopter video through calibration, detection–tracking, and a Monte-Carlo procedure for lane assignment—an acknowledged hard problem at high altitude—and, tellingly, benchmarked the result against NGSIM US-101, finding more plausible speed and acceleration distributions and lower internal and platoon consistency errors than the long-standing de facto standard [18]. Fonod et al. pushed the template to production scale in Songdo, South Korea: a detector tailored to high-altitude bird’s-eye imagery, a stabilization scheme that masks moving vehicles out of image registration, orthophoto- and master-frame-based georeferencing across multiple drone viewpoints, and dimension estimation, applied to roughly 12 TB of video over twenty intersections during four days. The outputs—about 700,000 trajectories plus more than 5000 annotated images with some 300,000 vehicle instances—were validated against a high-precision instrumented probe vehicle, and the entire pipeline was released as open source [19]. Berghaus et al. added a complementary validation pathway, projecting detections onto the road surface via three-dimensional camera calibration and cross-checking speeds and accelerations against induction loops and in-vehicle smartphone sensors, reporting deviations of roughly 0.45 m/s and 0.3 m/s^2^, respectively [20].

Two clarifications about the methodological composition of the chain are warranted, because a reading confined to the tables can obscure them. First, learned models, predominantly deep neural networks, are not one option among several in this literature; they are its substrate. Every detector of Section 2.1 [9,10,11,13,14], the appearance embeddings of DeepSORT and StrongSORT [15,16], the tailored detector and multi-stage training of the Songdo pipeline [19], the segmentation networks that populate the static layer (Section 3.2), the crack-segmentation network and the quantized onboard detector of Section 3.3, and the detection front end of automated conflict diagnosis (Section 5.3) are learned models, and the benchmark that institutionalized aerial detection [12] was built to train and evaluate exactly such models, so the pavement study cited in Section 3.3 is representative rather than exceptional; the gradient-boosted correction used to denoise aerial kinematics (Section 5.1) is a learned but non-neural exception, and the histogram-of-oriented-gradients detector with a support vector machine in the pedestrian–vehicle conflict study of Section 5.3 is the classical one. Second, evolutionary and metaheuristic optimizers, including genetic algorithms, are essentially absent from the perception chain itself, where gradient-based training has displaced them; they survive in the mission-planning layer that decides where, when, and for how long to fly, in the form of coverage, routing, and battery-aware scheduling problems, which Section 7.2 examines, and, outside the perception chain, in the calibration of behavioral and simulation models against observed trajectories, the use closest to Section 5.4. That division of labor is, in our reading, appropriate: perception is a supervised learning problem with abundant labels, whereas flight planning is a combinatorial problem under hard energy constraints, and the two are rightly evaluated by different criteria. The computational requirements and software availability that condition how widely the chain can be operated are also deferred to Section 7.2.

A related question is whether aerial trajectories can substitute for onboard inertial measurement in the study of vehicle dynamics. The evidence of this section suggests a division of roles rather than a substitution. Aerial extraction observes position directly, in a fixed external frame and for every vehicle in view, so speed and acceleration are obtained by differentiation and inherit amplified noise; the Songdo authors note that earlier aerial pipelines reported acceleration distributions with heavy tails reaching hundreds of m/s^2^ before stabilization was addressed, tuned their own smoothing against the onboard record of an instrumented probe vehicle, and report mean positional deviations below 1 m at seven of the nine intersections at which the probe vehicle was observed and mean speed differences within about 1 km/h at eight of them [19], while the off-ramp dataset reports agreement with in-vehicle smartphone sensors within roughly 0.3 m/s^2^ in acceleration [20]. An inertial measurement unit (IMU) measures body-frame accelerations and angular rates directly, at hundreds of hertz and without differentiation, but drifts in position and equips one vehicle at a time. The complementarity is therefore exact: aerial observation captures the population-level kinematics of interacting vehicles, which no fleet of instrumented cars can achieve, whereas IMU and satellite positioning resolve the high-frequency dynamics of a single vehicle, which no camera at 100 m or more can achieve. The validation designs of [19,20], in which an instrumented vehicle drives through the aerial field of view, are the practical form of that cooperation, and we regard a standing protocol of this kind, with probe vehicles carrying real-time kinematic (RTK) satellite receivers and IMU sensors through every campaign, as the most direct route to certifying the kinematic content of the dynamic layer.

Three observations synthesize this section, and Table 2 supports them. First, the field has quietly converged on a pipeline template—stabilize, detect, track, georeference, filter—so that methodological novelty increasingly lives in the joints between stages rather than in any single model. Second, the binding constraint has shifted from detection accuracy to validation discipline: of the pipelines reviewed, only a minority verify outputs against independent ground truth such as probe vehicles [19] or loop detectors [20], and until such verification becomes routine, headline accuracy claims should be read as internal consistency statements. Third, openness is emerging as a scientific variable in its own right; the release of full extraction code alongside data [19] converts a dataset from a static artifact into a reproducible instrument, and we regard this practice—still the exception—as the single most cost-effective norm the community could adopt.

## 3. A Hierarchical Framework for Road Feature Database Construction

This section is the conceptual center of the review and delivers the second and third contributions of Section 1.2. Section 3.1 introduces the three-layer framework and the co-registration discipline that binds the layers; Section 3.2 and Section 3.3 review how the static layer and its pavement condition sub-layer are currently built and how far reported accuracies can be trusted; Section 3.4 shows what the layers enable when consumed jointly; and Section 3.5, which the three-layer classification alone does not supply, sets out the construction methodology itself as a six-stage workflow with the key implementation issues at each stage. The dynamic layer is populated by the datasets and campaigns reviewed in Section 4 and Section 5 and is therefore examined there.

### 3.1. Three Layers and Their Co-Registration

**Figure 1:**
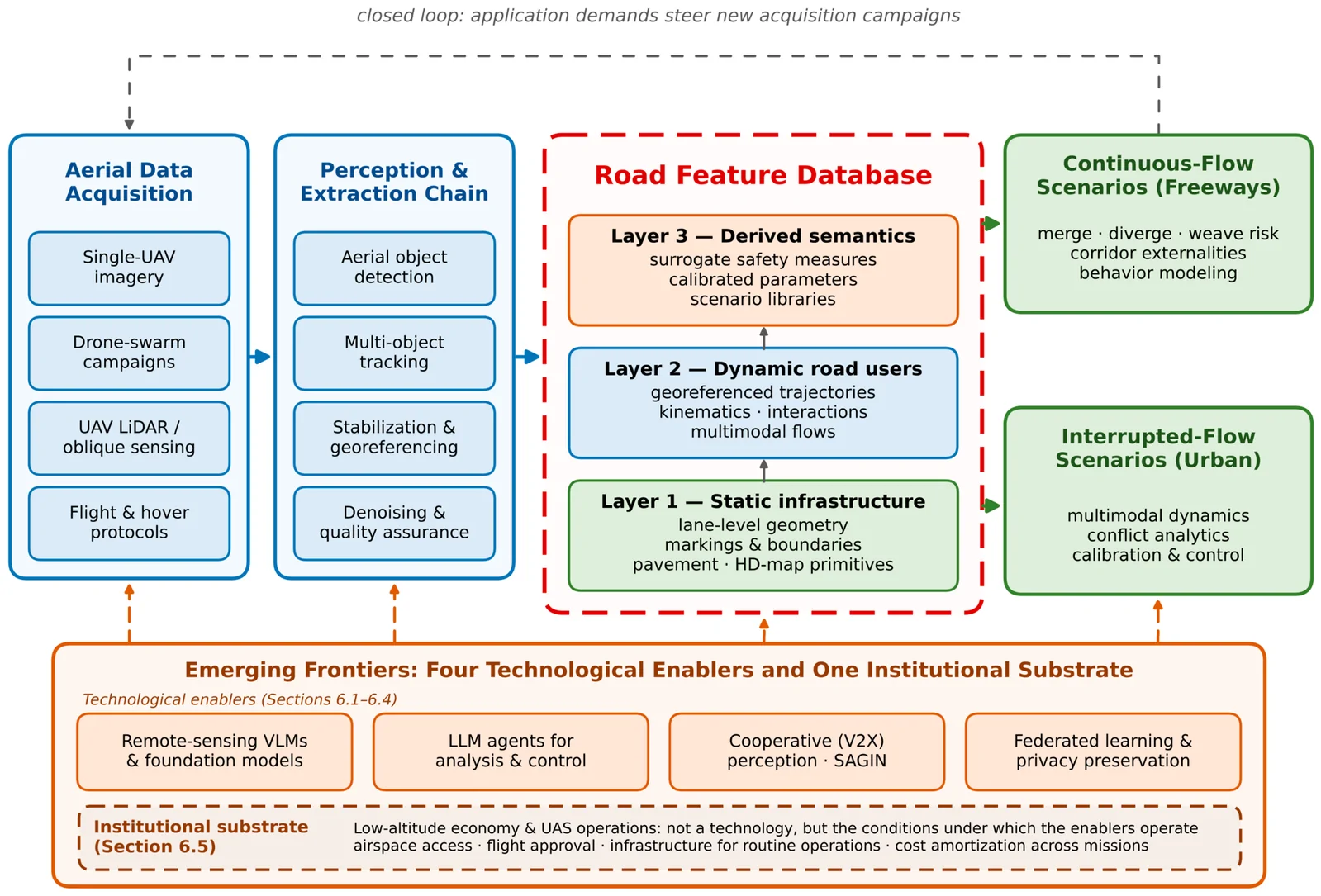
Reference framework for UAV-based road feature database construction. Aerial acquisition and the perception chain populate a three-layer database (static infrastructure features, dynamic road-user features, derived semantic features), which serves continuous-flow and interrupted-flow applications; emerging frontiers (Section 6: four technology clusters and, drawn separately as their institutional substrate, the low-altitude economy) act on all stages, and application demands close the loop by steering new acquisition campaigns. All remaining abbreviations are listed in the Abbreviations section.

Figure 1 organizes the literature of Section 2, Section 3, Section 4, Section 5 and Section 6 into a single construction framework. Aerial acquisition and the perception chain feed a database whose layers differ in temporal character: static infrastructure features change over months to years; dynamic road-user features change over fractions of a second; derived semantic features are computed, versioned interpretations of the other two. The framework’s essential discipline is co-registration—every trajectory should be resolvable against lane-level geometry, and every derived indicator should carry provenance back to the observations and algorithms that produced it. This is more than tidiness. Digital twin surveys repeatedly find that attention has concentrated on model creation while physical–virtual interaction lags [4], and that fidelity–computation balancing remains an open challenge while infrastructure health status is largely ignored [6]. In our reading, both frictions presuppose structured underlying data; a layered, co-registered database is precisely the missing contract between sensing and such downstream systems. The subsections below review how the static layer and its pavement sub-layer are currently built, how far reported accuracies can be trusted, and how a database is assembled in practice.

### 3.2. The Static Layer: Lane-Level Geometry, Markings, and Boundaries

Static-layer research splits along two methodological philosophies. The learning-centered philosophy treats feature extraction as semantic segmentation. A UAV LiDAR pipeline for lane-level road networks chains SegFormer road-area extraction, DBSCAN clustering with RANSAC plane fitting to strip pavement clutter, and PP-LiteSeg symbol extraction, and demonstrates robustness on an elevated inner-ring corridor in Shanghai’s Yangpu District—an environment where mapping vehicles are hampered by traffic and limited sensor range [21]. Capsule-based networks extract and classify markings from mobile laser scanning (MLS) clouds at 94.11% precision and an F1-score of 92.43%, holding a 3.42% average misclassification rate across urban roads, highways, and underground garages [22]. The geometry-centered philosophy instead encodes engineering priors. Constrained-RANSAC lane marking extraction with trajectory-based partitioning and statistical noise filtering attains F1-scores of 89–97% even on heavily worn stripes [23]; B-spline road-surface modeling with multi-frame fusion recovers boundary lines at an F1 of 0.9277 on KITTI [24]; and the scan-to-graph approach segments pavements into lanes, hard shoulders, and central reserves, fits marking centerlines in a Frenet frame, and stores the result as a hierarchical graph at roughly 90% mean intersection-over-union [25]. Where dense sensing is unavailable, expert-encoded reconstruction from map data alone has produced highway twins with centimeter-level average vertical deviation on the UK A1(M) [26].

A candid appraisal is warranted. The headline scores above—spanning F1 values from 0.89 to 0.97, alongside precision and mIoU figures of comparable magnitude—are obtained on different sensors, sites, and definitions of success, and are therefore not mutually comparable; the absence of a cross-sensor, cross-site benchmark for static road features is a conspicuous gap that mirrors what VisDrone resolved for aerial detection years ago [12]. More constructively, the two philosophies are converging: learned segmentation increasingly supplies candidates that geometric models regularize, and the Frenet-frame graph representation [25] is, in our assessment, the most database-ready output format yet demonstrated, because it encodes topology and geometry in a form that both trajectories and downstream twins can index. UAV-borne acquisition holds a further, pragmatic advantage over MLS in exactly the settings where static data are scarcest—congested or elevated corridors where mapping vehicles cannot operate freely [21]—which argues for UAV LiDAR and imagery as first-class, not auxiliary, sources for the static layer.

### 3.3. The Pavement Condition Sub-Layer

Pavement condition is the static layer’s most volatile component and, revealingly, its hardest perception problem. A systematic UAV imaging study covering platform assembly, flight settings, and a six-class distress dataset found that even the best of three mainstream detectors (YOLOv3, ahead of Faster R-CNN and YOLOv4) reached only 56.6% mean average precision [27]—a sobering figure beside the 90%-plus scores routine for lane markings, and a reminder that thin, low-contrast, texture-confounded cracks stress detectors in ways vehicles do not. Progress since has come from two directions. For onboard, real-time operation, the most instructive advance arrives from adjacent bridge-inspection work: the YOLOv6s-GRE line, developed for multi-class structural damage detection, combined an anchor-free architecture with a generalized feature pyramid and re-parameterized aggregation, then applied optimizer-based re-parameterization and partial quantization-aware training to gain 2.3 points of mAP50 while accelerating inference by 83.5% and cutting energy use by 79.7% [28]—a recipe that transfers directly to pavement imaging because it attacks the same endurance budget limiting UAV inspection of any linear asset. For accuracy, image–LiDAR fusion proved conditionally useful: adding elevation to RGB raised crack recall by about two points and improved sealed-crack segmentation by four to seven points across datasets, but fusing low-cost LiDAR intensity degraded precision, recall, and F-measure by roughly two, eight, and five points because of sensor noise [29]. The honest conclusion is that fusion is not a free lunch; its value is contingent on sensor quality and flight altitude, and database schemas should therefore record acquisition metadata alongside condition labels so that later users can judge which fused products to trust.

### 3.4. From Feature Layers to Digital Twins and Scenario Assets

The framework’s payoff appears when layers are consumed jointly. Graph-structured static geometry [25] and expert-reconstructed alignments [26] provide the skeleton that digital twins require; trajectory layers—reviewed next in Section 4 and Section 5—supply the twin’s dynamic content and, when curated as scenario libraries, feed the validation of automated driving systems, the very purpose for which the drone-dataset lineage was created [30]. Derived semantic features close the loop toward practice: surrogate safety indicators, calibrated parameters, and versioned analytics turn raw coverage into decisions. Table 3 consolidates the static-layer evidence; its most important column, in our view, is the last one, because it makes explicit how heterogeneous today’s outputs remain—rasters, point clouds, polylines, graphs—and thus how much standardization work Section 7 must still call for.

### 3.5. Construction Methodology: A Six-Stage Workflow and Its Key Implementation Issues

The three layers describe what a road feature database contains; they do not, by themselves, say how one is built. Reading the pipelines and campaigns of Section 2, Section 3, Section 4 and Section 5 as instances of a common procedure, we distill a six-stage construction workflow: (1) acquisition design, (2) perception and extraction, (3) georeferencing and co-registration, (4) quality assurance and uncertainty annotation, (5) schema design, storage, and provenance, and (6) release, governance, and maintenance. The stages are sequential in a first campaign but cyclic thereafter, because the application demands of Section 4 and Section 5 feed back into acquisition design (Figure 1). Table 4 pairs each stage with the decisions it forces, the most instructive practices in the reviewed literature, and the gap that remains; where the instructive practice belongs to a study introduced later in the review, the corresponding section is indicated rather than the reference, so that the numbering of references by first appearance is preserved.

Four issues in Table 4 deserve emphasis because they recur across otherwise unrelated pipelines. The first is the reference frame. A trajectory is only as reusable as the coordinate system it is expressed in, and the most mature release provides every point in three frames at once, namely the pixel coordinates of a reference orthophoto, a projected local Cartesian system, and geographic coordinates, together with the road section and lane identifiers that tie the dynamic layer to the static one [19]; that redundancy, rather than any single frame, is what makes later co-registration cheap. The second is temporal alignment: multi-drone campaigns are network-scale only if clocks are synchronized and session boundaries are recorded, because battery-driven rotations leave gaps that downstream users must be able to see. The third is provenance: derived features such as surrogate safety indicators should carry the algorithm version and thresholds that produced them, and condition labels should carry the acquisition metadata that governs whether a fused product can be trusted, which is the lesson of the image–LiDAR fusion results of Section 3.3 [29]. The fourth is quality metadata: a per-point visibility or confidence flag [19] costs almost nothing to store and changes what downstream models can legitimately conclude. None of these issues is technically hard; each is organizationally neglected, which is why Section 7 treats standards and metadata as the highest-leverage near-term investment.

## 4. Continuous-Flow Scenarios: Freeway Applications

Section 4 and Section 5 together deliver the fourth contribution of Section 1.2: they read the application literature through the two facility regimes of capacity analysis, continuous flow and interrupted flow, so that the data each regime demands can be compared. Freeways are where the drone-dataset movement began, and where the dynamic layer of Figure 1 is most mature. We organize this section around three questions: what corridor-scale observation now exists (Section 4.1); what merging, diverging, and weaving research it has enabled (Section 4.2); and how aerial snapshots relate to the fixed sensing that freeways increasingly carry (Section 4.3).

### 4.1. Corridor Observation and Freeway Trajectory Datasets

The highD dataset demonstrated the paradigm’s economics: 16.5 h of drone video over six German sites yielded 110,000 vehicles, 45,000 driven kilometers, and 5600 complete lane changes, expressly to support scenario-based validation of automated driving [30]. Its acknowledged blind spot—merging—motivated exiD, recorded at Autobahn entries and exits, which contributes 69,172 road users over more than 16 h of measurement and substantially more interactive scenarios, and whose authors articulate the recording method’s defining advantage: from the air, every road user remains visible free of vehicle-to-vehicle occlusion, while the traffic under observation proceeds uninfluenced [31]. Berghaus et al. extended coverage into regimes the family had lacked—an off-ramp and congested traffic—contributing 8648 trajectories over 87 min and roughly 1200 m, together with the loop and smartphone validation already discussed in Section 2.3 [20]. The instructive counterpoint is I-24 MOTION, which trades the drone’s flexibility for permanence: 276 pole-mounted cameras over 4.2 miles of Interstate 24 observe on the order of 230 million vehicle-miles annually and publish multi-day trajectory releases [32]. The comparison crystallizes the freeway data economy. Drones deliver deployment-free, geometry-agnostic, occlusion-free coverage but in episodes of tens of minutes per battery (a limit whose operational workarounds Section 7.2 examines); fixed instruments deliver continuity but at one heavily engineered site. Neither substitutes for the other, and a database-centric research program should treat them as complementary feeds into a common trajectory schema rather than as competing methods—a point to which Section 4.3 returns.

### 4.2. Merging, Diverging, and Weaving: Behavior and Risk

Ramp-related segments concentrate both crashes and the analytical value of aerial data, because the full spatial evolution of a mandatory maneuver becomes observable. Gu et al. built the template at interchange merges: a multilevel random-parameters logistic model of merging behavior estimated from UAV trajectories, coupled to TTC so that risk reflects behavior rather than proximity alone; merging speed, driver ability indicators such as lane-change confidence and lane-keeping instability, and merge location all shaped risk [33]. The connected-and-automated-vehicle (CAV) transition now complicates this picture. A spatiotemporal assessment framework combining negative-binomial conflict prediction with grid-based risk mapping, applied to a carefully calibrated microsimulation, predicts that as CAV penetration rises from 10% to 50%, risk hotspots migrate from the geometric bottleneck at the ramp nose to a dynamic interaction interface on the mainline, improving the merge area’s safety grade from D to A [34]. We read this result with measured enthusiasm: it is simulation-born, and precisely the kind of prediction that the empirical drone corpus of Section 4.1 is best placed to confirm or refute as mixed traffic materializes—an underexploited validation opportunity the two communities have yet to seize jointly.

Diverge and weave studies complete the picture. At exit ramp terminals, trajectories of 3259 vehicles across six sites revealed a design-relevant asymmetry: at taper-type deceleration lanes drivers begin braking on the freeway’s right lane, disturbing through traffic, whereas parallel-type lanes largely avoid this; drivers also adopt a single sustained deceleration rate, and ramp control features measurably shape diverging behavior [35]. On weaving segments, a lane change risk assessment index (LCRAI) was constructed by fusing exposure—via generalized TTC and an exposure-to-risk index with spatiotemporal overlap screening—with severity based on post-collision velocity change; applied to a Chinese expressway weave, it located the highest conflict frequency near the exit but the greatest integrated risk in the segment’s middle, with the final 100 m safest [36]. Taken together, these studies showcase the analytical dividend of trajectory completeness, but they also expose an indicator proliferation problem: TTC, generalized TTC, exposure indices, and composite scores each carry bespoke thresholds, and results are rarely transferable across sites. Databases can discipline this by shipping open, versioned implementations of surrogate measures with the trajectories themselves, so that risk estimates become recomputable rather than merely reported.

### 4.3. Corridor Externalities and Fixed-Infrastructure Complements

Trajectory databases repay their construction cost partly by serving purposes their builders never envisioned. A striking example computes traffic noise from drone-derived trajectories: treating each vehicle as a moving source under the CNOSSOS emission model, the study quantified noise at bus stops, correlated emissions with congestion at the microscopic level, and recovered clear macroscopic patterns tied to vehicle accumulation [37]—an environmental application that required no additional sensing whatsoever, only a sufficiently rich dynamic layer. Safety applications extend to facility types that fixed sensors serve poorly: at a toll plaza diverging area in Nanjing, trajectory extraction and an extended TTC showed that manual-toll vehicles carry the highest collision risk, that risk grows with proximity to toll lanes and surrounding traffic intensity, and that mixing electronic and manual collection elevates danger overall [38]. That finding acquires fresh relevance from the infrastructure side. A recent synthesis of 71 studies traces how China’s networked electronic toll collection (ETC) gantries have evolved from payment terminals into pervasive sensing nodes supporting travel time estimation, flow prediction, origin–destination inference, and safety analysis—while candidly identifying two binding limitations: spatial–temporal resolution constrained by gantry spacing, and real-time capability limited by transmission latency [39]. The complementarity is exact: gantries provide always-on, low-resolution coverage of the whole network; drones provide episodic, centimeter-and-decisecond resolution wherever a question arises. A common, co-registered database schema—Section 3’s central proposal—is the precondition for exploiting that complementarity rather than merely remarking upon it.

## 5. Interrupted-Flow Scenarios: Urban Applications

Interrupted-flow facilities—signalized and unsignalized intersections, roundabouts, and dense urban networks—multiply the entities, modes, and conflict mechanisms that a database must represent. This section reviews network-scale campaigns (Section 5.1), node-level datasets (Section 5.2), conflict analytics and design evaluation (Section 5.3), and the emerging closure of the loop from observation to calibration and control (Section 5.4).

### 5.1. Network-Scale Campaigns and Multimodal Dynamics

The pNEUMA experiment redefined the achievable scale: a swarm of ten drones hovering over 1.3 km^2^ of central Athens—more than 100 lane-kilometers and roughly 100 intersections—captured on the order of half a million trajectories across multiple days and released them as an open observatory of congestion [40]. What followed is, for this review’s thesis, the most instructive part. The pNEUMA Vision extension restored the imagery layer with vehicle annotations and azimuth attributes, separated stationary from non-stationary errors—showing the latter dominate the noise—and demonstrated that density-map counting can match detection-based counting while rendering congested queues more legibly in time–space diagrams [41]. A follow-up study by the original investigators with additional collaborators then developed a principled denoising pipeline, combining smoothing filters with gradient-boosted correction under adaptive regularization, explicitly to spare future users the manual thresholding that raw aerial kinematics otherwise require [42]. On the modeling side, the dataset enabled the first multimodal network-level analyses of their kind: mode-specific macroscopic fundamental diagrams in which each mode’s mean speed responds to the accumulations of all modes, an extended two-fluid formulation, and a trip-based model reproducing the network’s congestion evolution [43]; and a two-layer reading of arterial lane use in which the operative ‘active’ lanes diverge from the marked ones under frictions from bus and taxi stops and illegal parking, with powered-two-wheeler filtering documented empirically [44].

Two lessons generalize. First, pNEUMA is the clearest existing embryo of the database vision: a campaign that became an ecosystem, with imagery extensions, denoising toolchains, and modeling studies emerging through the original investigators’ continuing collaborations with additional researchers. Second, and more cautionary, the very existence of a substantial post hoc cleaning literature demonstrates that raw aerial output is not analysis-ready; quality assurance is a first-class layer, not an afterthought. The downstream stakes are rising as data-hungry forecasting matures—contrastive, multilayer graph architectures now explicitly model heterogeneity across temporal scales and spatial regions and outperform strong baselines across five benchmarks [45]—because such models will inherit whatever biases and artifacts the dynamic layer carries. A database that documents its own noise treatment is therefore not a luxury but a validity requirement for the learning systems built on top of it.

### 5.2. Node-Level Datasets for Intersections and Roundabouts

**Figure 2:**
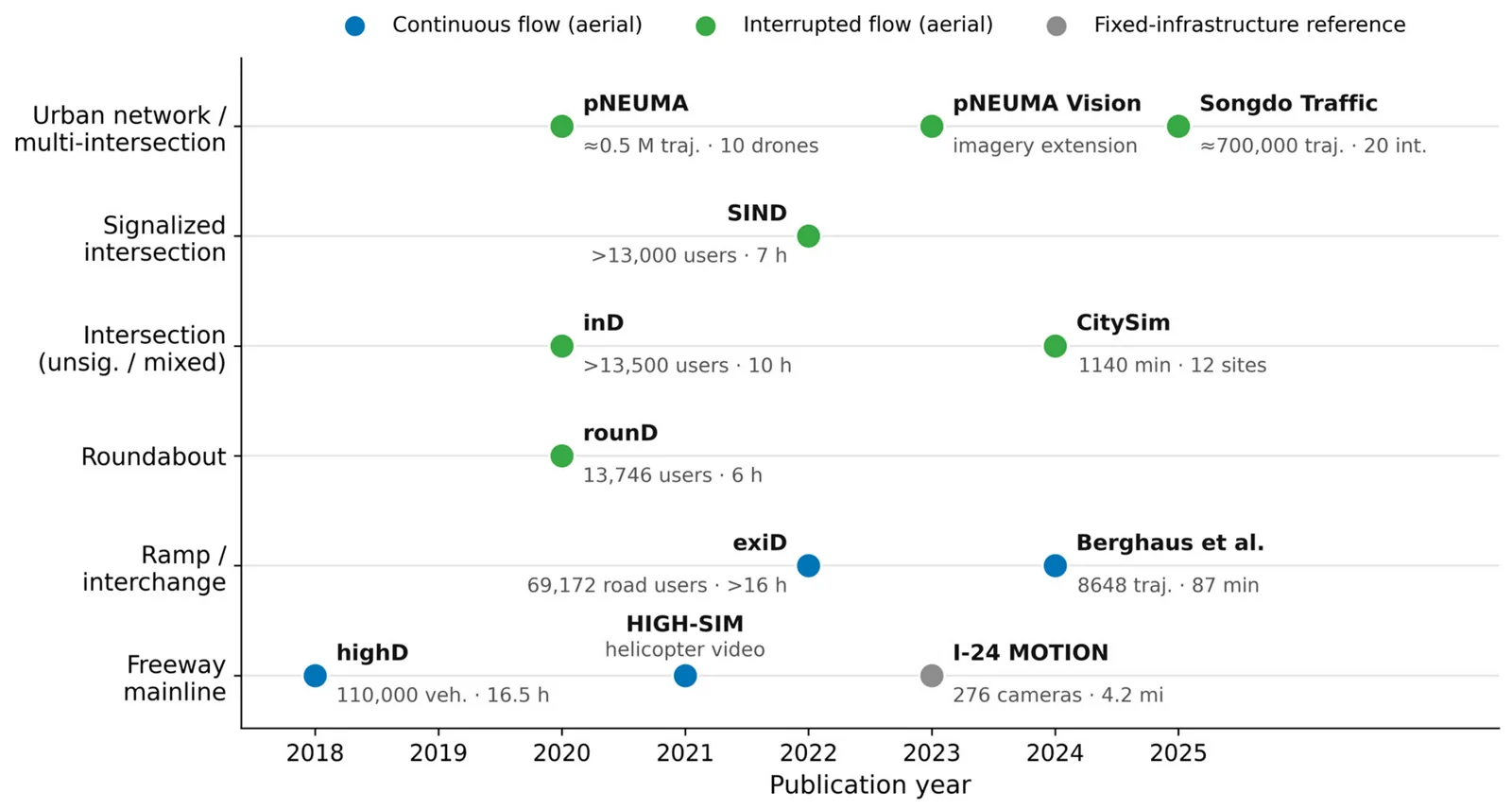
Landscape of trajectory datasets reviewed in Section 4 and Section 5, arranged by publication year and facility type: highD [30], pNEUMA [40], inD [46], rounD [47], HIGH-SIM [18], exiD [31], SIND [48], I-24 MOTION [32], pNEUMA Vision [41], Berghaus et al. [20], CitySim [49], and Songdo Traffic [19]. Annotations report each release’s scale in its own published unit (road users, trajectories, or recording duration; cf. Table 5); marker size is uniform and carries no quantitative meaning. The fixed-infrastructure I-24 MOTION instrument is included as a reference point. The unsignalized classification of the inD sites follows the dataset documentation (see Table 5); CitySim, which spans freeway segments and intersections of several control types, is placed on the mixed-intersection row.

The German drone-dataset family extended from freeways to nodes with inD—more than 13,500 road users, including substantial vulnerable road user (VRU) shares, over 10 h at four intersections, recorded from an angle that minimizes occlusion and without road users noticing the measurement [46]—and rounD, which contributed 13,746 road users over 6 h at three roundabouts, environments whose interaction density makes them a stress test for behavior planning [47]. SIND moved the frontier to a signalized, heavily multimodal context: a two-phase intersection in Tianjin yielding over 13,000 traffic participants of seven classes in 7 h, uniquely packaged with traffic light states, a high-definition map, motion parameters, and recorded signal violations, with VRUs constituting 62.6% of participants [48]. The CitySim dataset then prioritized safety analytics at breadth: 1140 min of drone video across twelve locations spanning freeway basic, weaving, and merge–diverge segments as well as signalized, stop-controlled, and control-free intersections, processed through a five-step pipeline with rotated bounding boxes, and shown—via minimum-TTC and post-encroachment-time distributions—to contain markedly more safety-critical events than comparable video datasets, alongside 3D base maps and signal timings for digital twin work [49]. Figure 2 situates these releases jointly with the freeway corpus of Section 4.1, and Table 5 consolidates their parameters.

Read jointly, Figure 2 and Table 5 support three assessments. Scale has advanced substantially, if unevenly: the largest aerial releases grew from 110,000 recorded vehicles in 2018 to roughly half a million trajectories in 2020 and about 700,000 by 2025, and, in per-intersection terms, node-level coverage expanded by roughly an order of magnitude—from more than 13,500 road users over four intersections in 2020 to approximately 700,000 vehicle trajectories across 20 intersections in 2025—though the reported units (road users versus vehicle trajectories) are not identical. Duration, however, has not kept pace: nearly all aerial releases remain snapshots of hours to days, so temporal phenomena such as day-to-day variability and seasonal effects stay outside the dynamic layer’s reach. Composition has improved more convincingly: the interrupted-flow branch now contributes VRU-rich, signal-aware, violation-inclusive content that the freeway branch never needed. Geography, however, remains concentrated in Germany, the United States, Greece, China, and Korea, and the family’s coverage of adverse weather and night conditions is essentially nil—biases that any claim of generality built on these data quietly inherits. Section 7.2 examines why this is so and what it would take to change it.

### 5.3. Conflict Analytics and Design Evaluation at Nodes

Intersection safety research traces a clear methodological arc across the reviewed period—from detection-centric automation toward inference-centric statistics. The ARCIS system marks the first pole: Mask R-CNN detection with reliability-aware tracking, rotated bounding rectangles derived from pixel masks for faithful vehicle footprints, and pixel-level post-encroachment time (PET) yielding automated identification of rear-end, head-on, sideswipe, and angle conflicts at a signalized intersection [50]. For the mixed traffic that dominates many Asian cities, the vehicle Safety Space concept inferred empirically the clearance envelope drivers maintain—using classical histogram-of-oriented-gradients (HOG) features with support-vector-machine (SVM) detection and Kalman tracking—and used it as a spatial conflict filter validated at intersections in Taipei, Bangkok, and Mumbai [51], an early and still instructive demonstration that automobile-centric conflict logic does not transfer unmodified to motorcycle-dense streams. The inference-centric pole has since matured rapidly: random-parameters logit models with heterogeneity in means and variances now estimate real-time motorcycle and non-motorcycle conflict propensity from drone trajectories, isolating speed, acceleration, lateral motion, conflict type, and time-to-green as drivers [52], while Bayesian Tobit formulations at the signal-cycle level show that ignoring temporal correlation and site heterogeneity materially degrades fit, with traffic volume, shock wave area and speed, queue length, and platoon ratio emerging as the operative predictors [53].

Two recent developments push the arc toward practice. A complete monitoring framework—drone operation design, state-of-the-art trajectory extraction, kinematic profiling, and TTC/PET-based analysis—was demonstrated at two busy signalized intersections in Manchester, localizing conflict-prone zones, attributing them to specific critical movements, and establishing an empirical link between speed and near-crash frequency [54]; this is, to date, the most persuasive end-to-end evidence that swarm-based safety diagnosis can be operational rather than exploratory. Complementing diagnosis with intervention, the dynamic straight-right lane (DSRL) concept re-times shared straight-and-right lanes through pre-signals, and its evaluation—the vehicle delay model validated in SUMO, with sensitivity analyses of the main parameters carried out in a MATLAB environment built on real intersection geometry—reports reduced delays for both movements, improved lane capacity, and fewer right-turn–pedestrian conflicts [55]. We highlight DSRL less for the specific design than for its evaluative logic: every quantity it optimizes—delay, capacity, pedestrian conflict frequency—is exactly what a mature intersection database computes routinely, which is to say that database-driven diagnosis and design-side innovation are already speaking the same language, even if the two studies rarely cite each other. The remaining soft spot in this whole line of work is validation against actual crash outcomes; surrogate-measure validity is still mostly assumed, and node-level databases that accumulate long-horizon observations are the only realistic path to testing it.

### 5.4. Closing the Loop: Calibration Toward Control

The quiet workhorse application of node-level aerial data is simulation calibration, because a single hover simultaneously exposes demand, throughput, processing rates, and performance. Demonstrations at signalized intersections and roundabouts show saturation flow rates being tuned until simulated queue dynamics and delays match observation, and critical gaps and move-up times likewise adjusted for roundabouts across three real-world cases [56]. This matters beyond simulation fidelity: calibrated models are the currency in which control systems and design interventions are appraised, so the calibration pathway is how the derived semantic layer of Figure 1 begins to influence infrastructure decisions. What is still missing—and what Section 6 examines—is the institutional and computational machinery for this loop to run continuously rather than campaign by campaign.

## 6. Emerging Frontiers: Four Technological Enablers and One Institutional Substrate

Section 2, Section 3, Section 4 and Section 5 exposed recurring frictions: static-layer extraction remains a set of bespoke pipelines, access to the data demands specialist skills, dynamic-layer observation is confined to single-viewpoint episodes, raw footage cannot circulate across institutions, and the economics of flying remain those of one-off research charters. Four technological currents address the first four of these frictions in turn: remote-sensing foundation models the bespoke pipelines, large language model (LLM) agents the specialist interface, cooperative air–ground perception the single viewpoint, and federated learning the immobility of raw footage. The fifth friction, the economics of flying, is not a technological problem, and the current that bears on it, the low-altitude economy, is not a technology. It is the regulatory, infrastructural, and economic substrate that determines whether any of the four technologies is ever operated routinely over public roads rather than in one-off research charters, and it is examined separately in Section 6.5 for that reason. This section examines each current not as a general survey but through a single lens: what it changes for the construction, maintenance, and use of road feature databases.

### 6.1. Remote-Sensing Vision–Language Foundation Models for the Static Layer

The static-layer pipelines of Section 3 share a structural weakness: each feature class—lane markings, boundaries, distress—requires its own trained extractor and its own annotation budget. Vision–language foundation models attack this at the root. RemoteCLIP, the first vision–language foundation model for remote sensing, converts heterogeneous annotations into a unified image–caption format through box-to-caption and mask-to-box conversion, deliberately folds UAV imagery into pretraining, and then serves zero-shot classification, retrieval, few-shot learning, and object counting from a single backbone; across 16 evaluation datasets it surpasses the previous state of the art by 9.14% mean recall on RSITMD and 8.92% on RSICD, and beats the CLIP baseline by up to 6.39% average zero-shot accuracy over 12 downstream datasets [57]. The significance for database construction is the amortization logic: semantics become a property of the foundation model rather than of each bespoke pipeline. Grounding and interaction are advancing in parallel. GeoChat—motivated explicitly by the observation that general-domain models return inaccurate or fabricated content on remote-sensing queries—accepts region inputs, grounds its answers in spatial coordinates, and demonstrates robust zero-shot captioning, visual question answering, scene classification, and referring detection [58]; RSGPT contributes the complementary data asset, a 2585-caption human-annotated corpus (RSICap) with scene- and object-level detail plus the RSIEval benchmark, arguing that small volumes of high-quality alignment data suffice to adapt large models [59]. Closest to this review’s concerns, SAM-Road adapts the Segment Anything Model to extract vectorized road network graphs—vertices from probability masks, edges from a lightweight transformer head—producing complete multi-square-kilometer graphs in seconds at accuracy comparable to RNGDet++ while running 40 times faster on the city-scale dataset [60].

Our assessment is enthusiasm with two caveats. First, the evaluation benchmarks on which these models are validated remain dominated by satellite-style nadir imagery; the oblique, low-altitude, motion-rich footage that traffic databases ingest constitutes a domain shift whose magnitude is simply unmeasured, because no traffic-grade aerial instruction-following corpus yet exists—an omission the field could remedy cheaply by building on the datasets of Table 5. Second, hallucination control through grounding, GeoChat’s central contribution, is not optional for engineering databases: a fabricated lane count is worse than a missing one. The pragmatic near-term recipe is therefore hybrid—foundation models for open-vocabulary semantics and network topology, retained specialist pipelines with quantified accuracy (Table 3) for geometry-critical layers.

### 6.2. LLM Agents as Query and Decision Interfaces

A database that only specialists can query underdelivers on its investment. TrafficGPT addresses exactly this interface problem by fusing LLMs with specialized traffic foundation models, yielding four capabilities with direct database relevance: viewing and processing traffic data for decision support, decomposing broad tasks into sequenced calls to specialized models, assisting human decision-making through natural-language dialog, and iterating on outputs through interactive feedback [61]. In the vocabulary of Figure 1, the ‘traffic foundation models’ it orchestrates are precisely the derived semantic layer, and the LLM becomes a conversational front end to the whole stack. One step further, LLMs have been cast as controllers rather than interfaces: an agent-development framework for adaptive signal control instantiated both a zero-shot chain-of-thought controller and a Generally Capable Agent that accumulates knowledge from environmental interaction, and the latter reduced halted vehicles by 48.03% while raising mean speed by 25.29% against fixed-time, gap-based, and delay-based baselines [62]. The caveat is equally instructive: the evaluation lives at a single simulated intersection in SUMO, so the evidence certifies reasoning capability, not deployment readiness. We draw a system-level conclusion from this pairing: LLM agents amplify whatever data substrate they stand on—grounded on a curated, uncertainty-annotated database they democratize access; ungrounded, they hallucinate fluently. The database is thus not made obsolete by the agent; it becomes the agent’s guardrail.

### 6.3. Cooperative Air–Ground Perception and Engineering Platforms

The dynamic layer reviewed so far derives from a single aerial viewpoint; cooperative perception replaces the viewpoint with a fabric. TUMTraf-V2X quantifies the dividend: 2000 labeled point clouds and 5000 labeled images from five roadside and four onboard sensors, 30k 3D boxes with track identities across nine categories—including violations, near-misses, overtaking, and U-turns—and a cooperative camera–LiDAR fusion model that gains +14.36 3D mAP over its vehicle-only counterpart [63]. The same fusion logic extends naturally upward to drones as mobile nodes of the sensing fabric. What cooperative sensing looks like at engineering scale, rather than dataset scale, is documented by a multi-intersection cooperative control platform for CAV environments: a three-layer architecture separating data access, middleware, and application; a graph-structured data model of network topology; fusion staged at parameter, feature, and decision levels; and functions—speed guidance, emergency-vehicle priority, blind-spot warning—operating across onboard units, roadside units, and control centers, with field deployments reporting delay reductions of roughly 10% at key intersections in central Guangzhou and vehicle identification accuracy above 99.9% in a Foshan tolling-integration test [64]. We note a convergence that neither literature has remarked upon: the platform’s layered data architecture and graph-based network model, arrived at from engineering practice, mirror the layered database framework this review distilled from research prototypes (Figure 1)—independent evolution toward the same design is itself evidence the design is sound. Sustaining such fabrics at scale ultimately rests on communications: the space–air–ground integrated network (SAGIN) agenda for 6G—terrestrial nodes, UAVs and high-altitude platforms, and low Earth orbit satellites unified with integrated communication and computing under high mobility and unbalanced traffic—defines the substrate on which continuous aerial database refresh would stream [65].

### 6.4. Federated Learning for Privacy-Preserving Database Federation

The multi-institution future implied throughout this review collides with an inconvenient fact: raw aerial footage captured at low altitude, obliquely, or with zoom may still contain identifiable detail, and agencies frequently cannot share it for legal and data-ownership reasons. Federated learning (FL) offers the standard resolution—train shared models where the data resides—and its ITS deployment surveyed to date spans object recognition, traffic management, and service provisioning, with privacy protection and scalability as the motivating properties [66]. The same survey is refreshingly candid about the residual frictions, and they map one-to-one onto the database problem: uneven data distributions that are not independent and identically distributed (non-IID)—two cities’ intersections differ in geometry, fleet mix, and behavior far more than two hospitals’ X-rays differ—limited storage and computation at the edge, which on UAV platforms is a hard constraint, and privacy and security exposures that FL narrows but does not eliminate, motivating complementary collaborative technologies [66]. Our reading is that FL’s realistic near-term role in this field is model federation rather than data federation: perception backbones and denoising models trained across institutions, while trajectory-level releases continue to rely on the privacy advantages that aerial viewpoints naturally afford [41]. Federated construction of a shared static layer—where geometry is not privacy-sensitive but jurisdiction is—remains, to our knowledge, entirely unexplored, and may be the lower-hanging fruit.

### 6.5. The Low-Altitude Economy as an Institutional Substrate

The low-altitude economy (LAE) is treated here as an institutional substrate rather than as a technology, because what it changes is not any step of the extraction chain but the conditions under which the chain is operated: airspace access, flight approval, infrastructure for routine operations, and the amortization of costs across missions. Every campaign reviewed in Section 4 and Section 5 was flown as a bespoke research operation; the LAE is what changes that cost structure. Architecturally, low-altitude intelligent transportation is being designed as a cyber–physical system with a layered structure and an explicit whole-lifecycle management chain from design and construction through operation and management, resting on the Internet of Things, 6G, artificial intelligence, and low-altitude geographic information systems [67]—exactly the institutional machinery a standing road-observation service would inhabit. The supply side is responding to policy: a double machine learning analysis of China’s low-altitude airspace opening policy on listed aviation-manufacturing firms from 2003 to 2023 finds a robust positive effect on innovation—validated with difference-in-differences and generalized random forest designs—concentrated most strongly in drone enterprises, state-owned firms, and coastal regions [68]. Demand aggregation is visible in adjacent missions: UAV–ground collaborative emergency delivery has matured from single-aircraft operations toward multi-modal collaborative systems, with hybrid optimization, multi-UAV cooperation, AI enhancement, and real-time adaptation identified as the governing trends, and regulatory integration, adverse-weather adaptability, cybersecurity, human–machine interfaces, cost–benefit assessment, and standardization as the persisting obstacles [69]—a challenge list that overlaps almost verbatim with the data-collection agenda of Section 7, which is precisely the point: shared corridors, vertiports, traffic management, and certification regimes amortize across missions, and policy frameworks are being drafted along coordinated dimensions from latency and autonomy through regulation, acceptance, and equity [2]. The dependency also runs in reverse: safe low-altitude routing over urban corridors presupposes accurate, current models of the roads and activities beneath—which is to say, the road feature database is simultaneously a customer of LAE infrastructure and a supplier of its ground truth.

## 7. Cross-Cutting Synthesis, Operational Preconditions, and a Research Agenda

### 7.1. Cross-Cutting Synthesis: Methodological Families, Data Sources, Evaluation Metrics, and Technological Waves

Section 7, together with Section 6, delivers the fifth contribution of Section 1.2. Section 2, Section 3, Section 4, Section 5 and Section 6 organized the literature by database layer and facility regime. Read across those divisions, the reviewed studies are connected by three things they share: the methods they use, the data they build on, and the metrics by which they judge success. Table 6 makes those connections explicit for eleven research streams, and three observations follow from it.

First, the evaluation metrics form a chain, not a set of independent scores. Detection is judged by average precision, tracking by identity preservation, extraction by deviation from reference sensors, the static layer by F1 or mean intersection-over-union, safety analytics by the distributions of surrogate measures, and operations by delay, queue length, and capacity. Each metric is computed on the output of the stage before it, so error propagates along the chain in a direction that the individual studies do not measure: an identity switch inside a weaving segment becomes a spurious lane change, a spurious lane change becomes a false conflict, and a false conflict becomes a misallocated countermeasure. The chain is this review’s most concrete argument for the database as the unit of evaluation, because only a co-registered database exposes the whole chain to inspection at once.

Second, the dynamic-layer literature is more interconnected than its citation practice suggests, because a small number of research lineages account for a large share of it. The German drone-dataset family (highD, exiD, inD, and rounD) carries one recording method and one validation purpose across freeways, ramps, intersections, and roundabouts [30,31,46,47]; the pNEUMA lineage grew from a single campaign into an ecosystem of imagery extensions, denoising toolchains, network-level models, arterial and noise studies, an operational safety-monitoring framework, and a production-scale successor in Songdo [19,37,40,41,42,43,44,54]; and the surrogate-safety lineage carried one analytical template, UAV trajectories coupled to time-to-collision or post-encroachment time, from interchange merges to toll plazas, weaving segments, automated conflict diagnosis, and a purpose-built safety dataset [33,36,38,49,50]. Methods, data, and metrics travel together inside these lineages, which is why results within a lineage are comparable and results across lineages usually are not, and why the cross-region benchmarks called for in Table 7 matter.

Third, the field has developed in three waves, and the waves explain the trend directions in the last column of Table 6. A first wave (2017–2019) carried ground-level detection and tracking into aerial use, with the deep association metric of DeepSORT [15] as its representative method, established the drone benchmark that defined the aerial regime (VisDrone, first organized in 2018 and consolidated in [12]), and produced the drone-based naturalistic freeway dataset that set the template for the field [30], early UAV-based safety analyses at merges and toll plazas [33,38], and robust lane-marking extraction from mobile laser scanning [23]. A second wave (2020–2022) multiplied and diversified datasets, from a network-scale swarm [40] to nodes [46,47,48], helicopter corridors [18], and merging scenarios [31], automated conflict analytics [50,51], adapted detectors to drone imagery [13,14], and gave the static and pavement layers their learned and geometric extractors [22,24,26,27]. The third wave (2023–2026) is the one this review argues the field is now in: validation discipline and production scale [19,20,41,42,49,54]; open-vocabulary semantics and conversational interfaces [57,58,59,60,61,62]; cooperative, federated, and institutionally embedded operation [63,64,65,66,67,68,69]; and a recent benchmark, one of the few designed for severe weather and low visibility, that measures how aerial vehicle detection degrades under rain, fog, snow, and night [70]. The direction of travel is consistent across streams, from single-purpose outputs toward reusable, validated, and maintained assets, which is the database vision stated in other words.

### 7.2. Operational Preconditions for Sustained Observation

Between the technical possibilities of Section 6 and the research agenda of Section 7.3 stand five operational questions that any agency contemplating a standing road-observation service will ask first, and that the reviewed literature answers only in passing: how continuous coverage is maintained when a battery lasts minutes, what weather does to sensing and to flight, who permits the flight, what happens to the privacy of the people filmed, and what computing and software the extraction requires. We collect the evidence here because the answers condition every challenge cluster in Table 7.

Endurance and battery logistics. Multirotor endurance on the platforms used in the reviewed campaigns is measured in tens of minutes, and no campaign has escaped that limit by flying longer; all have escaped it by flying more. The Songdo campaign structured each day into ten synchronized sessions of roughly 30 min, each followed by a battery replacement in a fixed 40 min cycle, and accumulated 400 flights over four days in this way [19]; the pNEUMA swarm launched together, began recording once every drone was on station, returned its drones to their landing points as batteries ran low, and repeated the cycle in successive sessions to cover a 2.5 h morning peak, so that no recording exists during the changeovers [40]; and the Manchester monitoring framework, whose goal was long-duration recording, had pilots land each drone as it reached a minimum battery level, exchange the battery, and relaunch, and states explicitly that battery limits shaped the experiment design [54]. Three lessons follow. Substitution of a discharged drone by a charged one is at present a human scheduling problem, solved by redundancy in aircraft and batteries and by battery exchange on the ground rather than by exchange in flight: where sessions are synchronized the resulting gaps are common to the whole swarm [19,40], and where drones are rotated one by one the gaps are distributed across the fleet [54]. The gaps are consequential, since a collision in the Songdo campaign occurred during a battery swap and was therefore not recorded, although its aftermath was [19], and they must be documented as metadata (Table 4). The scheduling itself is an optimization problem, and it is here rather than in perception that evolutionary and metaheuristic methods, including genetic algorithms, have their established role, in coverage path planning, routing, and energy-aware multi-UAV coordination [71]. Finally, the standing observatories anticipated in Section 6.5 will need what research campaigns lack, namely automated docking with battery exchange or tethered power supply, so that continuity becomes a property of the infrastructure rather than of the pilots.

Weather and environmental limits. Adverse weather acts on the database twice, on the flight and on the sensing. On the flight, the Songdo campaign lost its first planned day to strong winds that exceeded the drones’ operational limits, together with rain and fog [19], and adverse-weather adaptability is among the persisting obstacles listed for collaborative UAV missions in adjacent fields [69]; wind also degrades stabilization, which is why the extraction pipelines of Section 2.3 treat platform motion as a first-class error source. Low temperatures compound the endurance problem: environmental chamber tests of a battery-powered UAV found that low temperature significantly degraded both flight performance and battery performance, with the platform unable to operate normally below −25 °C, whereas heat of up to 60 °C left flight performance largely unaffected but shortened battery life and reduced discharge capacity [72], so the already short hover endurance shrinks further in exactly the seasons that the datasets of Table 5 never cover. On the sensing side, the effect has now been quantified for aerial vehicle detection: the SWUAV benchmark, published in this journal and presented by its authors as one of the few aerial datasets built explicitly for severe weather and low visibility, contains 18,195 aerial images with 236,392 vehicle instances across twelve weather and illumination conditions, including heavy rain, dense fog, blizzards, and backlit night scenes; its authors report that a strong real-time detector reaches only 43.9% AP on it, that their weather-adapted architecture raises this to 46.9%, and that models lose more than 50 percentage points of AP50 (average precision at an intersection-over-union threshold of 0.50) when transferred from it, without adaptation, to a drone dataset and a hazy-scene dataset [70]. The implication for database construction is direct: the near-zero adverse-weather coverage noted in Section 5.2 is not an accident of scheduling but the joint product of flight restrictions, endurance loss, and perception degradation, and a database that records weather as acquisition metadata (Table 4) is the precondition for ever measuring the bias this induces.

Airspace access and regulation. Every aerial campaign in Section 4 and Section 5 was flown under a national permission regime, and the regimes share a structure even where their details differ: small UAVs are assigned to a default envelope defined by an altitude ceiling, visual line of sight, and exclusion from controlled airspace, with case-by-case approval or waivers for anything beyond it. The reviewed campaigns comply visibly with such envelopes: the Songdo drones held 150 m, reduced to 140 m where collision risk required, to remain within South Korean rules, and were flown by certified pilots [19]; the Manchester campaign used professional pilots at 120 m [54]; and the inspection literature catalogs the permission, liability, and privacy conditions that determine where and how often flights are possible [3]. What the low-altitude economy changes, as Section 1 and Section 6.5 argued, is the transition from exceptional permission toward routine, infrastructure-supported access along the regulatory and acceptance dimensions structured in [2] and the management chain envisaged in [67]. Until that transition is complete, airspace approval belongs in the acquisition metadata of a database alongside altitude and weather, because it constrains where and when the dynamic layer can be refreshed.

Privacy of road users. The privacy question has a geometric answer and an institutional one. Geometrically, the nadir view from 100 to 150 m yields ground sampling distances of several centimeters per pixel, which is why a bounding-box center error of 2.21 pixels in the Songdo detector corresponds to 12.2 cm [19]; at that resolution faces are not resolvable and license plates span too few pixels to be read, so the aerial viewpoint carries a privacy advantage that ground-level cameras lack, a point already made in the pNEUMA extension work [41]. Institutionally, the drone-dataset family releases trajectories rather than the recorded video [30,31,46,47]; the pNEUMA release did the same and added imagery and vehicle annotations only later, in a separate extension [40,41]; and the Songdo release ships trajectories with brief video samples, the extraction code, and, as a separate vision dataset, 5419 annotated frames selected for detector training [19]. The release of raw campaign video across agencies remains the exception, which is the sharing friction that federated learning addresses at the model level [66] and that legal analyses list among the binding conditions of operation [3]. For database construction, the practical rule that emerges is graded release: derived layers and trajectories openly, imagery under annotation and access control, raw video not at all, with the privacy screening step recorded as part of provenance (Table 4, stage 6).

Computational requirements and software. The pipelines reviewed here separate into an offline extraction regime and an onboard real-time regime, with different budgets. Offline extraction at campaign scale is dominated by detection at high input resolution: the Songdo pipeline ran its detector at 1920 × 1920 pixels over roughly 12 TB of 4K video, reports its computational timings on a workstation with a 24-core desktop processor (Intel Core i9-13900KF), one high-end consumer graphics card (NVIDIA GeForce RTX 3090), and 64 GB of memory, and chose classical rather than learned feature matching for stabilization partly because no annotated data were available to fine-tune learned matchers and partly to avoid inference on a graphics processing unit (GPU) at that data volume [19]. Tracking is comparatively cheap, since a motion-centric associator runs at more than 700 frames per second on a central processing unit (CPU) [17], and detection throughput depends strongly on input resolution: the weather-adapted detector of [70] reaches 323 frames per second on a single consumer graphics card, but at an input of 640 × 640 pixels, roughly one ninth of the pixels processed per frame in the Songdo pipeline. A workstation-class GPU therefore appears adequate for offline extraction at campaign scale, although no reviewed study reports an end-to-end processing time for a full campaign, and the binding cost is annotation and quality assurance rather than computation. Onboard real-time operation faces the opposite constraint of energy, and the quantized YOLOv6s-GRE line, with an 83.5% gain in inference speed and a 79.7% reduction in energy use, shows the recipe of anchor-free design, re-parameterization, and quantization-aware training [28]. On software, three tiers coexist: open-source research pipelines released with their data, of which the Songdo extraction, stabilization, and optimization codes are the most complete example [19]; commercial turnkey extraction services, such as the DataFromSky service that processed the pNEUMA footage [40]; and the bespoke laboratory code behind most application papers, which is rarely released. The commercial tier yields usable trajectories without in-house expertise but at the price of undisclosed processing, which is why the reproducibility norm advocated in Section 2.3 favors the first tier for database construction.

The five answers share a pattern: each is at present supplied by the discipline of individual research teams, and none is yet supplied by infrastructure, standards, or metadata. That is the sense in which they are preconditions rather than research challenges, and it is why stages 1 and 6 of Table 4 ask for endurance windows, weather, airspace approval, and privacy screening to be recorded with the data.

### 7.3. Challenge Clusters and Research Agenda

The preceding sections each closed with targeted critique, and Section 7.1 and Section 7.2 have added a cross-cutting synthesis and an account of operational preconditions; this subsection consolidates all of these threads into six challenge clusters and pairs each with the enabling assets already present in the reviewed literature and the research directions we judge most productive. Table 7 provides the synthesis; three cross-cutting observations follow.

First, the clusters are coupled, and the coupling has a direction: C1 (persistence) feeds C2 (quality) with the long-horizon data needed to characterize error stability; C2 and C3 (standards) jointly gate C4 (federation), because institutions will only federate around artifacts whose quality and format they trust; and C5 (generalization) and C6 (intervention) are downstream of all four—no foundation model escapes its training distribution, and no control loop should close over uncertain data. The practical implication is sequencing: standards and quality metadata are the highest-leverage near-term investments precisely because everything else queues behind them.

Second, the field’s evaluation culture lags its engineering culture. Detector and tracker benchmarks are mature (Section 2), yet there exists no shared benchmark for the database product itself—no reference site where competing extraction-to-schema pipelines can be scored on geometric accuracy, semantic completeness, temporal freshness, and uncertainty honesty. The datasets of Table 5 are the raw material for exactly such a benchmark, and constructing one is organizationally cheap relative to its coordinating value. Third, incentives require attention: campaign datasets earn citations, whereas curation, harmonization, and maintenance—the activities Section 5 and Section 6 identify as bottlenecks—largely do not. Data-descriptor publication venues and funder mandates for maintained infrastructure, both visible in adjacent fields, are the obvious instruments; without them, the one-campaign-one-paper equilibrium documented in Section 5.2 will persist by default.

## 8. Conclusions

This review set out to organize a decade of UAV-based road observation research around a database-centric thesis: that the field’s durable output is not any single detector, campaign, or case study, but structured, layered, reusable representations of road infrastructure and the traffic it carries. In pursuing that thesis it has made the five contributions stated in Section 1.2: a sensing-oriented appraisal of the aerial perception chain that judges detection, tracking, stabilization, and georeferencing by downstream trajectory quality and by validation against reference sensors (Section 2 and Table 2); a three-layer reference framework that binds static infrastructure, dynamic road-user, and derived semantic features through co-registration and provenance (Figure 1); a six-stage construction workflow with the key implementation issues, instructive practices, and remaining gaps at each stage (Table 4); a regime-based comparison of freeway and urban applications with a consolidated landscape of twelve trajectory dataset releases (Figure 2 and Table 5); and a cross-cutting synthesis of eleven research streams, four technological frontiers, the low-altitude economy, and five operational preconditions, distilled into six challenge clusters with priority directions (Table 6 and Table 7). These elements distinguish the review from the surveys compared in Table 1, which treat the UAV as a measurement instrument, organize the field by task, or start from the consumers of road data and treat its supply as given.

The evidence assembled supports four conclusions. First, the perception chain has crossed the threshold of sufficiency: modern aerial detection and tracking, stabilized and georeferenced with documented accuracy, can populate a dynamic layer whose fidelity is validated against reference sensors rather than assumed. Second, the hierarchical framework of Figure 1—static geometry, pavement condition, and dynamic behavior bound by common georeferencing—is not an aspiration but a reading of convergent practice, visible independently in research prototypes and engineering platforms alike. Third, the two flow regimes monetize the database differently and complementarily: continuous-flow facilities yield capacity, risk, and externality analytics from vehicle trajectories alone, while interrupted-flow environments force—and reward—multimodal, signal-aware, conflict-rich representations. Fourth, read across layers and regimes, the literature is connected by a chain of evaluation metrics along which error propagates, by a small number of research lineages within which methods, data, and metrics travel together, and by three successive technological waves whose common direction is from single-purpose outputs toward reusable, validated, and maintained assets (Section 7.1).

For sensing research, these conclusions carry a practical corollary: the frontier of aerial traffic sensing has moved from the detector to the chain. Altitude and ground sampling distance, stabilization, synchronization across aircraft, georeferencing, and validation against reference sensors now determine whether a flight yields engineering-grade data, and the sensing problems that remain hard—pavement distress, adverse-weather and night operation, and endurance—are those for which no amount of downstream analysis compensates. The six-stage workflow of Table 4 is offered as the specification against which new sensing platforms, sensors, and processing chains for road observation can be designed and reported.

What remains is less a technology gap than an infrastructure gap. The four frontier technologies of Section 6 lower the marginal costs of semantic extraction, query access, sensing coverage, and institutional sharing, and the low-altitude economy lowers that of flight operations, but none of them, individually or jointly, substitutes for the unglamorous work Table 7 prioritizes: accuracy standards, shared schemas, governance templates, database-product benchmarks, and incentives for maintenance, together with the operational preconditions of endurance, weather, airspace, privacy, and computation that Section 7.2 sets out. The transition this review anticipates is from databases as by-products of research campaigns to databases as maintained scientific infrastructure—continuously refreshed through low-altitude operations, federated across institutions, queried in natural language, and trusted enough to sit inside control loops. The literature assembled here suggests that every component of that transition already exists somewhere in isolation; the research agenda is their integration.

The review has limitations of its own. It is narrative rather than systematic and favors archival journals and widely adopted benchmarks over gray literature and commercial systems (Section 1.1); its framework and workflow are distilled from the reviewed studies rather than tested in a new campaign; and its assessment of the emerging frontiers necessarily rests on early evidence. Validating the framework and workflow against a purpose-built reference site, as proposed in Section 7.3, is therefore the most direct test of the contributions made here.

## Figures and Tables

**Table 1:** Positioning of this review relative to the related surveys cited in Section 1, with the five contributions of Section 1.2 as the dimensions of comparison. Yes indicates a principal focus; Partial indicates a topic treated incidentally or from a different angle; No indicates a topic not treated.

Survey	Primary Scope and Organizing Principle	Aerial Perception Chain Assessed for Database Construction (Contribution 1)	Layered Database Framework and Construction Workflow (Contributions 2 and 3)	Freeway Versus Urban Regime Contrast (Contribution 4)	Emerging Frontiers, Cross-Cutting Synthesis, and Research Agenda (Contribution 5)
Obaid et al. [1]	UAVs coupled with AI for traffic and safety analyses (more than 315 studies, 2009–2024), organized by facility type and analysis task	Yes (AI models for detection, tracking, and parameter extraction)	No (the UAV is treated as a measurement instrument)	Partial (facility-specific data collection protocols)	Partial (challenges and opportunities)
Dilek and Dener [8]	Computer vision applications in ITS, organized by task (monitoring, incident detection, pricing, condition assessment)	Partial (ground and aerial vision, task by task)	No	No	Partial
Adibfar et al. [3]	Technical and legal challenges of UAVs in bridge monitoring and inspection	Partial (inspection sensing)	No	No	Partial (legal, privacy, and liability conditions)
Yan et al. [4]	Digital twin enabling technologies for road engineering, organized by lifecycle stage and enabling technology	Partial (condition sensing as a twin input)	Partial (data perception and virtual model creation)	No	Yes (digital twin technologies)
Ge and Qin [5]	Digital twin ITS, systematically reviewed by architecture and enabling technology	Partial (cooperative perception as an enabling technology)	Partial (data layer of the twin architecture)	No	Yes (fusion, cooperative perception, edge computing, federated learning)
Chang et al. [6]	Digital twins in transportation infrastructure: enabling technologies, applications, and challenges	Partial (sensing as an enabling technology)	Partial (data acquisition; fidelity–computation trade-offs)	No	Yes
This review	Construction of reusable road feature databases from UAV observations, organized by database layer and by facility regime	Yes (assessed by its effect on downstream trajectory quality and by validation against reference sensors)	Yes (three layers; six-stage workflow; key implementation issues)	Yes (Section 4 and Section 5; twelve dataset releases consolidated)	Yes (four technologies and the low-altitude economy as substrate; eleven research streams; six challenge clusters)

**Table 2:** Representative methods along the aerial perception chain and their relevance to road feature database construction.

Method	Stage	Key Idea	Reported Performance	Relevance to Aerial Databases
DETR [9]	Detection	Set prediction with bipartite matching; removes anchors and NMS	On par with Faster R-CNN on COCO	Simplifies dense-scene post-processing
Swin Transformer [10]	Detection backbone	Hierarchical shifted-window attention, linear complexity	58.7 box AP on COCO	Scalable backbone for large aerial frames
YOLOv9 [11]	Detection	Programmable gradient information (PGI) + GELAN	Train-from-scratch rivals pre-trained SOTA on COCO	Strong accuracy–efficiency balance for onboard use
TPH-YOLOv5 [13]	Aerial detection	Extra head for tiny objects; transformer prediction heads; attention	39.18% AP on VisDrone2021 test-challenge (≈+7 vs. YOLOv5)	Directly targets small, dense drone-view objects
UAV-YOLO [14]	Aerial detection	Receptive-field-oriented redesign of YOLOv3	Improved small-object detection on UAV-view data	Lightweight adaptation path for UAV imagery
DeepSORT [15]	Tracking	Deep appearance association metric	−45% identity switches vs. SORT	Longer coherent tracks under brief losses
StrongSORT [16]	Tracking	Modernized DeepSORT; AFLink; Gaussian-smoothed interpolation	SOTA on MOT17/MOT20/DanceTrack/KITTI	Millisecond-cost plug-ins for track completion
OC-SORT [17]	Tracking	Observation-centric correction of Kalman drift	>700 FPS on CPU; robust to occlusion/nonlinearity	Motion-centric fit for tiny aerial targets
HIGH-SIM pipeline [18]	Extraction + QA	Monte-Carlo lane assignment; NGSIM benchmarking	More plausible speed/acceleration distributions than NGSIM US-101	Early template for validated aerial trajectories
Songdo pipeline [19]	Extraction + QA	Moving-object-masked stabilization; orthophoto georeferencing	≈700,000 trajectories; probe-vehicle validated; open source	Production-scale, reproducible database instrument

**Table 3:** Representative studies constructing the static and pavement-condition layers of road feature databases.

Study	Platform/Sensor	Target Feature	Method Core	Reported Performance	Output Form
Kong et al. [21]	UAV LiDAR	Lane-level road network	SegFormer + DBSCAN/RANSAC + PP-LiteSeg	Robust on elevated urban corridor (Shanghai)	Lane-level network
Ma et al. [22]	Vehicle MLS	Road markings	Capsule extraction + hybrid capsule classification	94.11% precision; F1 92.43%; 3.42% misclassification	Classified markings
Jung et al. [23]	Vehicle MLS	Lane markings (incl. worn)	Constrained RANSAC + association + Dip-test filtering	F1 89–97% across sites	Vectorized lanes
Xiong et al. [24]	Vehicle LiDAR	Road boundaries	B-spline road model + multiRANSAC + fusion	F1 0.9277 (KITTI)	Boundary polylines
Pan et al. [25]	Point cloud (highway)	Lanes/shoulders/reserves	Segmentation + Frenet-frame curve fitting	≈90% mIoU	Hierarchical graph
Jiang et al. [26]	Map data	Alignment, cross-section	Engineering-expertise reconstruction	Centimeter-level vertical deviation (A1(M))	Highway digital twin
Zhu et al. [27]	UAV camera	6-class pavement distress	Faster R-CNN/YOLOv3/YOLOv4 comparison	Best mAP 56.6% (YOLOv3)	Distress detections
Yang et al. [28]	UAV (onboard)	Multi-class bridge damage	YOLOv6s-GRE + RepOpt + partial QAT	+2.3 mAP50; +83.5% speed; −79.7% energy	Real-time detections
Elamin & El-Rabbany [29]	UAV camera + LiDAR	Cracks/sealed cracks	Residual U-Net on fused channels	RGB F 82.26%; elevation +2% recall; sealed +4–7%	Segmentation masks

**Table 4:** Six-stage construction workflow for UAV-based road feature databases, with the key implementation issues at each stage, representative practices in the reviewed literature, and remaining gaps.

Stage	Key Implementation Issues	Representative Practices in the Reviewed Literature	Remaining Gap
1. Acquisition design	Altitude sets the trade-off between ground sampling distance, field of view, and small-object detectability; session length is bounded by battery endurance; multi-drone campaigns require overlapping views and synchronized clocks; wind, rain, fog, and airspace rules bound the operating window	Ten-drone fleet at 140–150 m flying synchronized 30 min sessions in a fixed 40 min cycle that includes battery replacement, 400 flights over four days, one planned day canceled for weather [19]; helicopter platform recording a freeway corridor continuously for about two hours [18]; battery-bounded swarm rotations of the urban campaigns of Section 5.1 and Section 5.3	No community campaign protocol; endurance windows, weather, and airspace conditions seldom recorded as metadata
2. Perception and extraction	Detector adapted to the nadir view and to tiny objects; tracker chosen for motion-dominated, low-occlusion scenes; class taxonomy harmonized across training sources; class labels made consistent along each track	Small-object heads and receptive-field redesign [13,14]; motion-centric association [17]; multi-stage detector training on a consolidated class set and confidence-weighted label aggregation along each track [19]	Trajectory-level error reporting absent; identity switches not tied to their engineering consequences
3. Georeferencing and co-registration	Stabilization against platform motion; transformation from image coordinates to a fixed frame and then to geographic and local Cartesian coordinates; lane assignment and curvilinear referencing; consistency across drones, viewpoints, and sessions	Track stabilization with moving-object exclusion masks, a master-frame intermediary, RTK-surveyed ground control points, and trajectories delivered in orthophoto, local Cartesian, and geographic coordinates with section and lane identifiers [19]; three-dimensional camera calibration projecting detections onto the road surface [20]; Monte-Carlo lane assignment [18]; Frenet-frame graph representation of the static layer [25]	No shared convention for coordinate frames, lane identifiers, or timestamps across campaigns
4. Quality assurance and uncertainty annotation	Independent reference sensors; separation of stationary from non-stationary error; smoothing that preserves dynamics; per-point quality flags; accuracy reported as metadata rather than in prose	Probe-vehicle comparison with mean positional deviations below 1 m at seven of nine intersections and mean speed differences within about 1 km/h at eight of them, and a per-point visibility flag [19]; loop-detector and smartphone validation [20]; benchmarking against NGSIM [18]; the error taxonomy and denoising toolchain of the pNEUMA ecosystem (Section 5.1)	Accuracy audits confined to isolated releases; no standard accuracy metadata
5. Schema design, storage, and provenance	Keys linking each trajectory point to lane-level geometry; versioned derived indicators carrying algorithm provenance; acquisition metadata (altitude, sensor, weather, session) stored with condition labels; file granularity and identifier scope; heterogeneous output forms (Table 3)	Per-intersection, per-session files with ISO timestamps, class, dimensions, kinematics, and section and lane fields [19]; hierarchical graph of lanes, shoulders, and reserves [25]; condition labels whose trustworthiness depends on recorded sensor and altitude metadata [29]; layered engineering data architecture with a graph-structured network model (Section 6.3)	No community layered schema; provenance rarely machine-readable
6. Release, governance, and maintenance	Privacy screening of imagery; graded access; code released alongside data; versioning and update cycles; cross-institution federation; incentives for curation	Data and the complete extraction, stabilization, and optimization code released together under open licenses [19]; trajectory-only releases of the drone-dataset family (Section 4.1); ecosystem growth through imagery extensions and denoising toolchains (Section 5.1); federated model training across institutions (Section 6.4)	Maintenance uncredited and unfunded; federated construction of the static layer unexplored

**Table 5:** Representative trajectory datasets serving the dynamic layer of road feature databases, across continuous-flow and interrupted-flow facilities.

Dataset	Year	Facility/Setting	Scale	Distinctive Assets	Noted Limitations
highD [30]	2018	German freeway mainline (6 sites)	110,000 vehicles; 16.5 h; 5600 lane changes	Naturalistic trajectories for AD validation	Merging scenarios absent
HIGH-SIM [18]	2021	US freeway (helicopter video)	High-granularity corridor trajectories	Monte-Carlo lane assignment; NGSIM benchmarking	Helicopter platform; limited sites
exiD [31]	2022	Autobahn entries/exits	69,172 road users; >16 h	Highly interactive merging scenarios	Cars/trucks/vans only
Berghaus et al. [20]	2024	Freeway off-ramp with congestion	8648 trajectories; 87 min; ≈1200 m	Loop and smartphone validation (0.45 m/s; 0.3 m/s^2^)	Short duration; single site
I-24 MOTION [32]	2023	Instrumented freeway (fixed cameras)	276 cameras; 4.2 mi; ≈230 M veh-mi/yr	Continuous observation; multi-day releases	Single corridor; heavy infrastructure
pNEUMA [40]	2020	Athens urban network (10 drones)	≈0.5 M trajectories; 1.3 km^2^; ≈100 intersections	Open multimodal congestion observatory	Noise requiring post hoc treatment [42]
pNEUMA Vision [41]	2023	Athens (imagery extension)	Imagery + annotations + azimuth	Error taxonomy; density-map counting	Inherits campaign coverage
inD [46]	2020	4 German intersections	>13,500 road users; 10 h	VRU-inclusive, low-occlusion viewpoint	Unsignalized contexts
rounD [47]	2020	3 German roundabouts	13,746 road users; 6 h	Dense-interaction node scenarios	Limited duration
SIND [48]	2022	Signalized intersection, Tianjin	>13,000 participants; 7 classes; 7 h	Signal states; HD map; 62.6% VRU; violations	Single intersection
CitySim [49]	2024	12 locations, mixed geometries	1140 min of drone video	Safety-critical event richness; DT assets (3D maps, timings)	Heterogeneous site coverage
Songdo Traffic [19]	2025	20 intersections, Songdo, Korea	≈700,000 trajectories; 12 TB source video	Probe-validated; full open-source pipeline	Four-day campaign window

**Table 6:** Cross-cutting synthesis of the reviewed literature by research stream: dominant methodological family, data basis, evaluation metrics, and the direction of technological change over the review period.

Research Stream	Dominant Methodological Family	Data Basis	Evaluation Metrics	Studies	Trend Direction, 2017 to 2026
Aerial object detection (Section 2.1)	Convolutional and transformer detectors; small-object heads; receptive-field redesign	COCO; VisDrone drone imagery	AP, mAP	[9,10,11,12,13,14]	From generic detectors toward aerial-specific, onboard-deployable models
Multi-object tracking (Section 2.2)	Tracking-by-detection; Kalman motion models with or without appearance embeddings	MOT17, MOT20, DanceTrack, KITTI	Identity switches; aggregate tracking scores; frames per second	[15,16,17]	From appearance-heavy toward motion-centric, low-cost association
Extraction pipelines and quality assurance (Section 2.3 and Section 5.1)	Stabilize, detect, track, georeference, filter; reference-sensor validation; learned denoising	Campaign video with probe-vehicle, loop, smartphone, and ground-control references	Speed and acceleration deviation from references; reprojection error; consistency errors	[18,19,20,41,42]	From unvalidated outputs toward audited, open pipelines
Static layer extraction (Section 3.2)	Semantic segmentation of point clouds and imagery; geometric fitting (RANSAC, B-spline, Frenet-frame curves); expert reconstruction	UAV and mobile LiDAR; point clouds; map data	Precision, F1, mIoU; vertical deviation	[21,22,23,24,25,26]	From per-feature extractors toward topology-aware graph outputs
Pavement condition (Section 3.3)	Convolutional detection and segmentation networks; image–LiDAR fusion; quantization for onboard inference	UAV imagery with optional LiDAR	mAP, recall, F-measure; speed and energy	[27,28,29]	From accuracy alone toward accuracy–energy trade-offs
Freeway datasets and behavior–risk analytics (Section 4)	Naturalistic trajectory datasets; random-parameters and logistic models; surrogate safety measures; microsimulation	Drone, helicopter, and fixed-camera trajectories; ETC gantry records	Dataset scale; TTC and PET distributions; model fit; risk indices	[18,20,30,31,32,33,34,35,36,37,38,39]	From mainline observation toward ramp, weave, and mixed-CAV risk, and toward fusion with fixed sensing
Urban campaigns, node datasets, and conflict analytics (Section 5.1, Section 5.2 and Section 5.3)	Multi-drone campaigns; multimodal macroscopic models; random-parameters logit and Bayesian Tobit conflict models; end-to-end monitoring frameworks	Multi-drone urban trajectories with signal states, HD maps, and imagery	Trajectory counts; VRU share; conflict rates; PET and TTC; model fit	[40,41,42,43,44,45,46,47,48,49,50,51,52,53,54]	From single-node snapshots toward network-scale, signal-aware, VRU-inclusive releases
Calibration, control, and engineering platforms (Section 5.3, Section 5.4, Section 6.2 and Section 6.3)	Simulation calibration; LLM-based signal control; cooperative platforms with layered data architectures	Drone-observed queues and delays; SUMO; field deployments	Saturation flow, critical gap, delay, halted vehicles, identification accuracy	[55,56,62,64]	From calibration toward closed-loop control, with field evidence still scarce
Foundation models and agents (Section 6.1 and Section 6.2)	Vision–language pretraining; grounding; promptable segmentation; LLM orchestration of traffic models	Remote-sensing image–caption corpora; city-scale road graphs; traffic data streams	Zero-shot accuracy and recall; graph accuracy and speed; task decomposition quality	[57,58,59,60,61]	From per-task extractors toward open-vocabulary, promptable semantics
Cooperative sensing and federation (technological enablers, Section 6.3 and Section 6.4) and the low-altitude economy (institutional substrate, Section 6.5)	Camera–LiDAR fusion; SAGIN architectures; federated training; cyber–physical system design; policy evaluation	V2X datasets; multi-institution data; policy and firm panels	3D mAP; delay reduction; convergence under non-IID data; innovation effects	[63,64,65,66,67,68,69]	From single-viewpoint sensing toward sensing fabrics and institutionalized operations
Endurance, robustness, and mission planning (Section 7.2)	Synchronized battery-rotation protocols in multi-drone campaigns; environmental testing of battery-powered platforms; severe-weather detection benchmarks; metaheuristic path and coverage planning	Campaign session schedules and flight logs; severe-weather aerial image sets; environmental chamber tests	Recording continuity and session gaps; AP under adverse conditions; endurance against temperature	[19,40,54,70,71,72]	From clear-weather, single-session campaigns toward sustained, all-weather observation

**Table 7:** Consolidated challenge clusters for UAV-based road feature databases, with enabling assets identified in the reviewed literature and priority research directions.

Challenge Cluster	Manifestation in Reviewed Literature	Enabling Assets	Priority Directions
C1. Endurance and temporal coverage	Aerial releases span hours to days; day-to-day and seasonal dynamics unobserved (Section 4.1 and Section 5.2)	Swarm operations [40]; fixed instruments [32]; roadside gantry networks [39]; synchronized multi-drone session cycles [19,40]	Hybrid persistent observatories mixing aerial snapshots with continuous ground sensing
C2. Quality assurance and uncertainty	Non-stationary error dominance [41]; dedicated denoising pipelines required [42]; accuracy audited only in isolated cases [19,20]	Error taxonomies; reference-sensor validation protocols; open extraction pipelines [19]	Standardized accuracy metadata; uncertainty propagation into downstream models (cf. [45])
C3. Interoperability and schema	One-campaign-one-format fragmentation (Section 5.2); static–dynamic layers rarely co-registered	HD-map generation chains [21]; graph extraction at scale [60]; digital twin standards and challenge discourse [4,6]	A community layered schema; alignment with HD-map and twin formats; database-to-twin update loops (cf. [5])
C4. Privacy, governance, and federation	Raw footage unsharable across agencies; legal and regulatory ambiguity [3]; acceptance concerns [2]; airspace and privacy preconditions (Section 7.2)	Privacy advantages of aerial viewpoints [41]; federated learning deployments [66]	Federated static-layer construction; graded-access release regimes; governance templates
C5. Generalization and geographic bias	Site concentration in a handful of countries; near-zero adverse-weather and night coverage (Section 5.2); domain shift for foundation models [58]; weather-induced perception loss and flight restrictions (Section 7.2)	RS vision–language corpora and benchmarks [57,59]; multimodal, VRU-rich datasets [48,49]; severe-weather aerial detection benchmark [70]	Traffic-grade aerial instruction corpora; cross-region transfer benchmarks; synthetic augmentation via twins
C6. From observation to intervention	Surrogate-to-crash validity largely assumed (Section 5.3); control evidence simulation-bound [62]	Operational safety-diagnosis frameworks [54]; calibration pathways [56]; cooperative platform research and deployments [7,64]; database-evaluable designs [55]	Field pilots with database-in-the-loop control; long-horizon crash-outcome validation of surrogate measures

## Data Availability

No new data were created or analyzed in this study. Data sharing is not applicable to this article.

## Abbreviations

The following abbreviations are used in this manuscript:

AD	Automated driving
AI	Artificial intelligence
AP	Average precision
CAV	Connected and automated vehicle
CPU	Central processing unit
DSRL	Dynamic straight-right lane
DT	Digital twin
ETC	Electronic toll collection
FL	Federated learning
FPS	Frames per second
GELAN	Generalized efficient layer aggregation network
GPU	Graphics processing unit
HD map	High-definition map
HOG	Histogram of oriented gradients
IID	Independent and identically distributed
IMU	Inertial measurement unit
ITS	Intelligent transportation system
LAE	Low-altitude economy
LiDAR	Light detection and ranging
LLM	Large language model
mAP	Mean average precision
mAP50	Mean average precision at an intersection-over-union threshold of 0.50
mIoU	Mean intersection-over-union
MLS	Mobile laser scanning
MOT	Multi-object tracking
NMS	Non-maximum suppression
PET	Post-encroachment time
PGI	Programmable gradient information
QA	Quality assurance
QAT	Quantization-aware training
RepOpt	Re-parameterizing optimizer (RepOptimizer)
RS	Remote sensing
RTK	Real-time kinematic
SAGIN	Space–air–ground integrated network
SOTA	State of the art
SVM	Support vector machine
TTC	Time-to-collision
UAS	Unmanned aerial system
UAV	Unmanned aerial vehicle
V2X	Vehicle-to-everything
VLM	Vision–language model
VRU	Vulnerable road user
